# Prediction at the intersection of sentence context and word form: Evidence from eye-movements and self-paced reading

**DOI:** 10.3758/s13423-022-02223-9

**Published:** 2022-12-12

**Authors:** Simona Amenta, Jana Hasenäcker, Davide Crepaldi, Marco Marelli

**Affiliations:** 1 University of Trento, Trento, Italy; 2 University of Milan-Bicocca, Milan, Italy; 3 International School for Advanced Studies (SISSA), Trieste, Italy; 4 University of Erfurt, Erfurt, Germany

**Keywords:** OSC, Surprisal, Prediction, Sentence reading, Eye movements

## Abstract

A key issue in language processing is how we recognize and understand words in sentences. Research on sentence reading indicates that the time we need to read a word depends on how (un)expected it is. Research on single word recognition shows that each word also has its own recognition dynamics based on the relation between its orthographic form and its meaning. It is not clear, however, how these sentence-level and word-level dynamics interact. In the present study, we examine the joint impact of these sources of information during sentence reading. We analyze existing eye-tracking and self-paced reading data (Frank et al., 2013, *Behavior Research Methods, 45*[4], 1182–1190) to investigate the interplay of sentence-level prediction (operationalized as Surprisal) and word Orthography-Semantics Consistency in activating word meaning in sentence processing. Results indicate that both Surprisal and Orthography-Semantics Consistency exert an influence on several reading measures. The shape of the observed interaction differs, but the results give compelling indication for a general trade-off between expectations based on sentence context and cues to meaning from word orthography.

**Supplementary Information:**

The online version contains supplementary material available at 10.3758/s13423-022-02223-9.

## Introduction

One central issue in language processing is how we read and understand words in sentences. An increasing amount of research indicates that a key feature of language comprehension is prediction (for a review, see Kutas et al., 2011); when reading a sentence, we expect upcoming words based on preceding words. For example, when reading “It was windy so the boy went out to fly a . . .”, readers commonly expect “kite” as sentence completion. The time we need to read a word has been found to depend on the amount of new or (un)expected information that it conveys (e.g., Frank, 2013; Smith & Levy, 2013; for a review, see Staub, 2015).

However, research on sentence reading often neglects that each word has its own recognition dynamics in spite of the context it is embedded in. While the most obvious effects of word length and frequency are sometimes assessed or at least controlled in sentence reading experiments (see Staub, 2015), the dynamics of how readers get from orthography to meaning, which have been identified in single word processing studies, are usually not taken into account. Orthographic strings can be more or less good cues for their meanings and this impacts on the ease of processing (Marelli & Amenta, 2018; Marelli et al., 2015). Hence, understanding a word in a sentence can gather from at least two different sources—namely, the sentence context and the word internal dynamics. The motivation of the present study was to bring together those two aspects that have been studied detached from each other and jointly investigate their dynamics when reading words in sentences.

The idea underlying the influence of sentence context onto word reading is that comprehension incrementally unfolds word by word. Hence, the words that have been read so far determine the extent to which a certain continuation is expected. Different measures have been used to quantify this. Early work on predictability has capitalized on cloze probability—that is, how likely a certain word is chosen by participants as a sentence continuation (Taylor, 1953). More recent work has increasingly used computational models to derive different metrics of predictability. One of the most commonly used ones is surprisal (e.g., Demberg & Keller, 2008; Frank, 2013; Frank et al., 2015; Hale, 2001; Monsalve et al., 2012). Surprisal captures the degree to which a word is unexpected given the preceding sentence context (e.g., Hale, 2001; Levy, 2008). Studies have shown that surprisal is an important factor influencing reading: reading times increase with increasing surprisal values (e.g., Boston et al., 2008; Boston et al., 2011; Demberg & Keller, 2008; Fossum & Levy, 2012; Frank & Bod, 2011; Frank et al., 2013; Mitchell et al., 2010; Monsalve et al., 2012; Roark et al., 2009; Smith & Levy, 2008). In particular, reading a word with higher surprisal has been found to increase early reading time measures, especially gaze duration (Aurnhammer & Frank, 2019; Smith & Levy, 2013), but also first fixation and regression-path duration (Lowder et al., 2018). These studies all indicate that the predictability of a word from the sentence context plays an important role for the word’s processing.

In the investigation of visual word recognition, many factors have been studied that influence the time it takes to process a word. Most of these factors are related to word form properties (e.g. length, number of orthographically similar words), lexical properties (e.g., word frequency) or semantic features (e.g., concreteness, valence, semantic richness). While these factors are highly informative of word processing and reliably predict recognition times, they mostly refer to a single linguistic level of analysis at a time (e.g., either form or semantics), but do not capture the interplay between these levels, which is crucial for reading. After all, orthography is the starting point of the process, while meaning is the endgame of comprehension. Recently, an attempt has been made at capturing the relationship between the orthographic form of a word and the ease with which it gives way to the activation of a specific meaning. Marelli et al. (2015) suggested that the time it takes to identify a word is influenced by the consistency, throughout the lexicon, between the orthographic form of a word and its semantics. For example, every time the orthographic string *widow* is encountered in the lexicon, even if embedded in other words (e.g., *widower, widowhood, widowed*), it points consistently to the meaning of WIDOW, as all words sharing this string of letters also share the core semantics. By contrast, the string *whisk* does not consistently point to a unique meaning as other words sharing this string have deviant semantics, such as *whisker* and *whiskey*. Marelli and colleagues hence proposed that words of the first type are good cues for their meaning, while the words of the second type are not. The degree of consistency of this form–meaning mapping was quantified by Marelli et al. (2015) and Marelli and Amenta (2018), with a measure termed Orthography-Semantics Consistency (OSC).^1^ OSC has been shown to influence visual word recognition—words with higher scores of OSC are recognized faster (Marelli & Amenta, 2018; Marelli et al., 2015)—and its effect holds across different word-recognition tasks (e.g., Amenta et al., 2020; Amenta et al., 2017) and against strong baselines including, for example, morphological family size, word length, and frequency. The OSC effect can be seen as the relative ease with which readers are able to form an expectation concerning the word semantics on the basis of its orthography.

In the present study, we bring together sentence context and word orthography as distinct, but potentially interacting sources of meaning activation. We test the hypothesis that, during natural sentence reading, two dynamics can influence processing: (a) expectations concerning the word based on the preceding sentence context, and (b) expectations concerning the meaning of the word on the basis of its specific orthographic form. To assess the interplay between two levels at which expectation can unfold, we analyze the eye tracking data provided by Frank et al. (2013) in Experiment 1 and self-paced reading times, from Frank et al. (2013) in Experiment 2. Sentence-based word predictability was operationalized in terms of surprisal (Frank et al., 2015; Hale, 2001; Levy, 2008) and orthography-based semantic activation was captured by OSC (Marelli & Amenta, 2018).

Surprisal can be thought of as a *horizontal* source of information for word meaning, being generated and continuously updated for any upcoming word as the sentence unfolds. OSC, by contrast, can be thought of as a *vertical* cue for word meaning that kicks in at the moment when the specific word is encountered and/or its orthographic form enters the visual word identification system. This distinction into two orthogonal dimensions parallels the concept of syntagmatic and paradigmatic relationships in linguistics, whereby a syntagmatic relationship involves sequences of units and a paradigmatic relationship involves mutually exclusive alternatives of units. Syntagmatic and paradigmatic effects have been shown to interact in studies on speech production (e.g., Kuperman et al., 2007; Lõo et al., 2022). It is quite unexplored, however, what the dynamics are between the orthogonal measures of surprisal and OSC in sentence reading. Based on their unique influence in previous studies, we suppose that they both have an effect on reading. There are no theoretical reasons that allow us to have strong hypotheses about whether they are additive or interactive, or even the shape of a possible interaction. Hence, in this sense, our study is exploratory. In the General Discussion, we will take up the theoretical implications of the actual pattern that we found as well as how alternative findings could have been interpreted in order to evaluate how our findings help to get a better sense of mechanisms involved in sentence reading.

## Experiment 1

### Methods

#### Data

The eye-tracking analyses are based on the publicly available reading time data by Frank et al. (2013). These authors provide a collection of eye tracking data from 43 participants (27 female, *M*_Age_ = 25.8 years) reading 205 independent English sentences, not including any violations or experimental manipulations and thus representing natural reading. Sentences were presented individually in a single line on the computer display and both eyes were tracked with the EyeLink II system (SR Research; see Frank et al., 2013, for details on material and procedure). As dependent variables for our study we consider four measures of reading times: first-fixation duration, gaze duration, right-bounded time, and regression-path time.

First-fixation time is defined as the duration of the first fixation on a word that has been fixated more than once (Bertram, 2011; also referred to as first-of-many fixation duration) and is generally considered a measure of early processing (e.g., Falkauskas & Kuperman, 2015; Schmidtke et al., 2018). Gaze duration is defined as the sum of all fixations on a word before moving the eyes away from it (Bertram, 2011). This metric has been taken as a measure of word access and is thus at the center of our analyses. Right-bounded time and regression-path time are taken from Frank et al. (2013). Both measures are considered to reflect later stages of processing: right-bounded time is the sum of all fixations on the target word before leaving it rightward; regression-path time is right-bounded time plus all the time spent on previous words during regressive eye-movements.

As a measure of sentence context information, we used Surprisal. Surprisal is based on the assumption that sentence processing is incremental and predictive: after reading *w*_*1*_*, w*_*2*_*, w*_*3*_ *. . . w*_*t-1*_, the system has estimated a probability distribution P(w_t_|w_1_ . . . w_t-1_). At this point, the identity of the word *w*_*t*_ is still unknown and is considered a random variable. The Surprisal value of the random variable *w*_*t*_ is defined as the negative of the logarithm of the probability of *w*_*t*_ given *w*_*1*_ *. . . w*_*t-1*_, or, in other words, the probability of the next word given the sentence. In mathematical terms, Surprisal is defined as:

1 $$\textrm{Surprisal}\left({\textrm{w}}_{\textrm{t}}\right)=-\log\ \textrm{P}\Big({\textrm{w}}_{\textrm{t}}\mid {\textrm{w}}_1\dots {\textrm{w}}_{\textrm{t}-1}\Big).$$

In particular, we adopted the recurrent neural network (RNN) surprisal measure as the best performing estimate from Frank et al. (2015)^2^ for the same set of sentences as was used in the eye tracking.

In order to have a measure of word internal dynamics (form–meaning mapping), we used OSC. OSC is computed as the frequency-weighted average semantic similarity between the meaning of a word and the meanings of all the other words that contain it, including the target itself (i.e., its orthographic relatives). OSC is defined as:

2 $$OSC(t)=\frac{\ {\sum}_{x=1}^k{f}_{r_x}\ast \cos \Big(\overrightarrow{t},\overrightarrow{r_x}\Big)}{\sum_{x=1}^k{f}_{r_x}},$$

where *t* is the target word, *r*_*x*_ each of its *k* orthographic relatives, and *f*_*rx*_ their corresponding frequencies (see Marelli et al., 2015, and Marelli & Amenta, 2018, for details). The semantic similarity between the target and its relatives is obtained by computing the cosine proximity *cos(t, r*_*x*_*)* between their corresponding word embeddings in a semantic space (Mikolov et al., 2013). OSC generally ranges from 0^3^ to 1: lower values correspond to less consistency, and higher values correspond to more consistency. For the present study, we retrieved OSC values from Marelli and Amenta (2018; see above) for each word in the eye-tracking dataset.

#### Analysis

Eye-tracking data were analyzed through generalized additive mixed models (GAMMs; Wood, 2006). First, we excluded data for which OSC values were not available from Marelli and Amenta (2018; 41.3%), and words for which OSC was equal to 1 (2.1%; following Marelli & Amenta, 2018). Next, data points with fixation durations shorter than 50 ms or gaze durations longer than 1,200 ms were excluded (38.9%). The final dataset included 28,005 data points.

The interaction between surprisal and OSC of the fixated words was modeled in nonlinear terms through tensor products including OSC and surprisal. Word length, log-transformed frequency, and position in the sentence were included in the model as linear terms.^4^ Random effects for subjects and items were modeled through splines (as in Feldman et al., 2015). Once the model was fitted, results were checked through model criticism (Baayen, 2008) by removing data points with particularly deviant residuals (more than 2.5 standard deviations) and refitting the model.

## Results

Results of the analysis on gaze durations (deviance explained: 16.4%) are reported in Table 1. We observed a significant nonlinear interaction between Surprisal and OSC (*p* < .0001). The inclusion of nonlinear terms was justified by a goodness-of-fit test: the fit of the model with the tensor product was significantly higher than the one obtained when the interaction was modeled in linear terms (*F* = 2.88, *p* = .0024). The nonlinear interaction held against model criticism, that is it remained significant (*F* = 5.04; *p* < .0001) when the model was refitted after removing data points with residuals exceeding 2.5 standard deviations.

**Table 1:** Summary of the model fit to the log-transformed gaze durations

	Estimate	Std. Error	*t* value	Pr(>|t|)	*t* value after model criticism	Pr(>|t|) after model criticism
Intercept	5.363	0.067	80.02	<.001	80.08	<.001
Length	0.006	0.007	0.91	.036	0.80	.422
log-Frequency	−0.001	0.004	−0.26	.079	−0.35	.728
Position in sentence	−0.007	0.001	−7.11	<.001	−11.42	<.001
	edf	Ref.df	*F*	*p* value	*F* after model criticism	*p* value after model criticism
te(OSC, Surprisal)	11.54	13.49	4.36	<.001	5.04	<.001
s(Subject)	41.25	42.00	66.87	<.001	82.01	<.001
s(Word)	284.89	352.00	5.53	<.001	7.04	<.001

te() denotes a tensor smooth; s() denotes a thin plate regression spline.

**Fig. 1:**
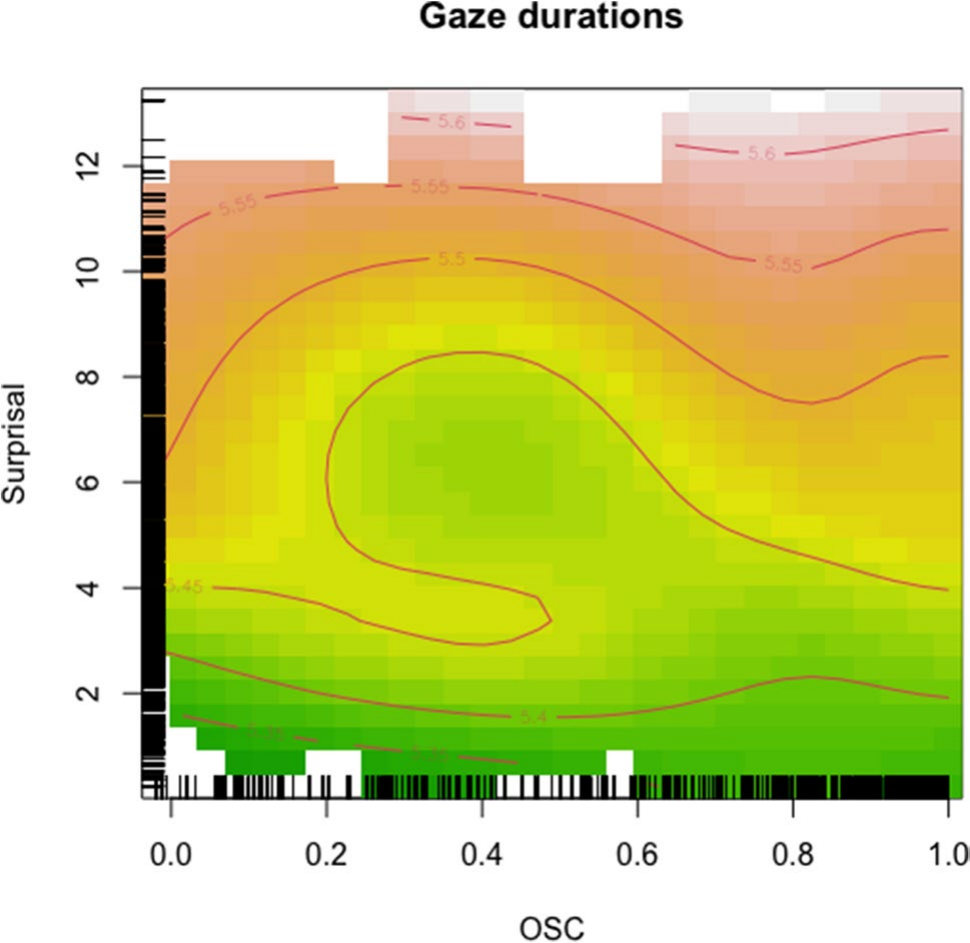
Tensor product smooth for the interaction of OSC (*x*-axis) and Surprisal (*y*-axis). Color shades indicate different log-transformed gaze durations, with green shades indicating shorter gaze durations and red shades indicating longer gaze durations. Rugs indicate distribution of data points. (Color figure online)

Figure 1 represents the interaction between OSC (*x*-axis) and Surprisal (*y*-axis). Gaze durations (log-transformed) are represented by different color shades, where green indicates shorter and red longer gaze durations. The figure indicates an effect of Surprisal: when Surprisal is particularly low (<3, lower part of the plot) or particularly high (>10, upper part of the plot) gaze durations are very short or very long, respectively, with no role for OSC. However, in its mid-range, the impact of Surprisal is modulated by the word’s OSC: The general Surprisal effect is confirmed only when OSC has extreme values (either low or high), whereas in OSC mid-range (roughly from 0.2 to 0.6) a boost in gaze time can be observed. In other words, at mid-range levels of both OSC and Surprisal the target item is easier to process, leading to shorter gaze durations.

The analysis on first fixation durations (deviance explained 13.2%) was based on 3,739 data points (data points for words receiving a single fixation were excluded; see Marelli & Luzzatti, 2012). On first fixation durations, the nonlinear interaction between OSC and Surprisal was not supported in terms of model fit, that is, the amount of explained variance by a model including the nonlinear term vis-à-vis a model with a linear interaction was not significant (*F* = 0.22, *p* = .6867). When modeled in linear terms, we did not find a significant effect for the interaction of interest (*t* = −1.279; *p* = .2011). We thus reran the model without the non-significant interaction, finding that only the simple effect of Surprisal (*t* = 2.63; *p* = .0085) remained significant: the higher the degree of Surprisal, the longer the first fixation on the target word. The effect held against model criticism (*t* = 2.69; *p* = .0071). Full details of this model are reported in Table 2.

**Table 2:** Summary of the model fit to the log-transformed first fixation durations

	Estimate	Std. Error	*t* value	Pr(>|t|)	*t* value after model criticism	Pr(>|t|) after model criticism
Intercept	5.134	0.059	87.74	<.001	95.54	<.001
Length	−0.010	0.006	−1.57	.116	−2.75	.006
log-Frequency	0.004	0.003	1.32	.187	0.75	.451
Position in sentence	0.005	0.002	2.56	.011	0.60	.597
OSC	0.009	0.031	0.29	.772	0.66	.507
Surprisal	0.009	0.003	2.56	.009	2.69	.007
	edf	Ref.df	*F*	p value	*F* after model criticism	*p* value after model criticism
s(Subject)	36.69	42.00	10.71	<.001	14.50	<.001
s(Word)	24.49	344.00	0.08	.081	0.23	<.001

s() denotes a thin plate regression spline.

The analyses of right-bounded time (deviance explained: 17.2%) and regression-path time (deviance explained: 13.3%) led to results paralleling those for gaze durations: In both, we found significant nonlinear interactions between OSC and Surprisal (*p* < .0001), that were supported in terms of goodness-of-fit when compared to corresponding models including linear characterizations of the interaction (*p* < .0001), and held against model criticism (*p* < .0001). Details concerning the results of the analyses are reported in Table 3 and 4.

**Table 3:** Summary of the model fit to the log-transformed right-bounded time

	Estimate	Std. Error	*t* value	Pr(>|t|)	*t* value after model criticism	Pr(>|t|) after model criticism
Intercept	5.394	0.072	74.46	<.001	74.70	<.001
Length	0.009	0.007	1.30	.195	1.28	.201
log-Frequency	−0.001	0.004	−0.20	.843	−0.41	.681
Position in sentence	−0.007	0.001	−6.88	<.001	−11.16	<.001
	edf	Ref.df	*F*	*p* value	*F* after model criticism	*p* value after model criticism
te(OSC, Surprisal)	12.78	14.85	5.07	<.001	5.97	<.001
s(Subject)	41.26	42.00	68.48	<.001	86.14	<.001
s(Word)	286.16	352.00	5.79	<.001	7.21	<.001

te() denotes a tensor smooth; s() denotes a thin plate regression spline.

**Table 4:** Summary of the model fit to the log-transformed regression-path time

	Estimate	Std. Error	*t*-value	Pr(>|t|)	*t*-value after model criticism	Pr(>|t|) after model criticism
Intercept	5.45	0.090	60.44	<.001	61.51	<.001
Length	0.006	0.009	0.63	.526	0.86	.390
log-Frequency	0.001	0.005	0.25	.803	−0.25	.802
Position in sentence	−0.001	0.001	−0.85	.396	−7.06	<.001
	edf	Ref.df	*F*	*p* value	*F* after model criticism	*p* value after model criticism
te(OSC, Surprisal)	14.72	16.76	4.21	<.001	3.91	<.001
s(Subject)	41.00	42.00	48.01	<.001	67.99	<.001
s(Word)	278.50	352.00	4.79	<.001	6.79	<.001

te() denotes a tensor smooth; s() denotes a thin plate regression spline.

**Fig. 2:**
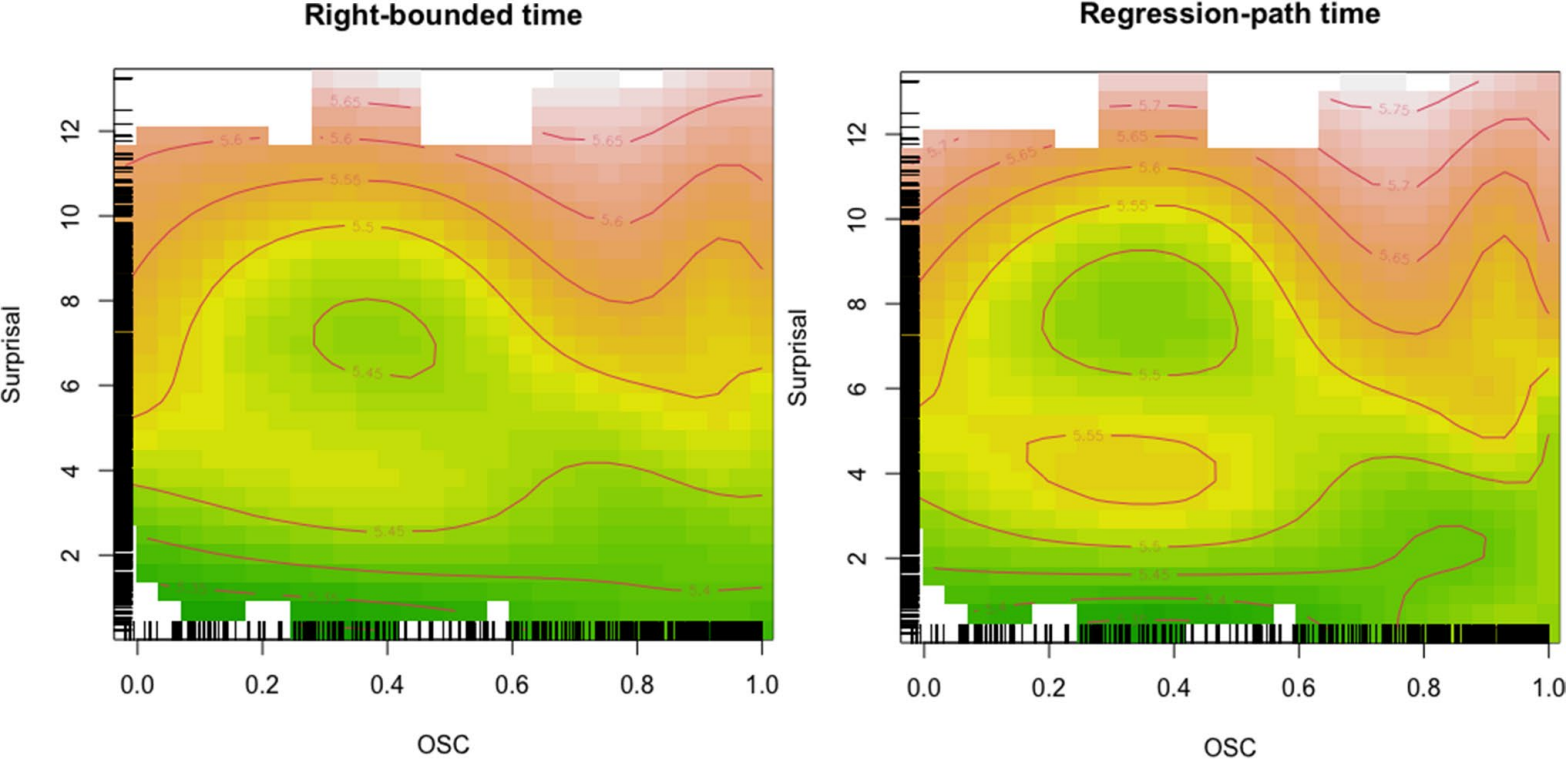
Tensor product smooth for the interaction of OSC (*x*-axis) and Surprisal (*y*-axis). Color shades indicate different log-transformed right-bounded times (left panel) and regression-path times (right panel), with green shades indicating shorter times and red shades indicating longer times. Rugs indicate distribution of data points. (Color figure online)

The nonlinear interactions between OSC and Surprisal for right-bounded time and regression-path time are represented in Fig. 2. The pattern is very similar to the one for gaze durations, with a general effect of Surprisal (with high Surprisal leading to longer fixation times) and a boost in looking times when both Surprisal and OSC are at mid-range level.

### Discussion

Experiment 1 showed that sentence context as a horizontal cue for word meaning and word orthography as a vertical cue for word meaning jointly affect eye movements in natural sentence reading. In gaze durations, right-bounded times, and regression-path times, we found Surprisal and OSC to interact. The results confirm our hypothesis that expectations based on the preceding sentence context and expectations based on a word’s specific orthographic form both exert an influence on reading; and they do so in an interactive way.

The observed interaction between Surprisal and OSC in the three eye movement measures (gaze durations, right-bounded times, and regression-path times) is nonlinear. While we did not have strong predictions on the shape of such interaction, we will make an attempt at interpreting this nonlinear interaction, with the goal of formulating hypotheses to be tested in the future.

Looking at gaze durations as an exemplary measure, we see that the pattern is dominated by short gaze durations in the central area of Fig. 1. Words in this region of the parameter space are mid-way on both the Surprisal and the OSC scales—they are somewhat surprising, but not extremely so, and their form points to their meaning, but without constraining it too much. In words that are highly predictable (i.e., low Surprisal) gaze durations are very short, as represented by the green strip at the bottom of Fig. 1. These words, along with their meanings, are easy to anticipate, and therefore it makes sense that OSC does not play a big role (as represented by the very little color modulation that one encounters moving horizontally from lower to higher OSC level in this region of the graph). This is because the reader can easily recognize the word without engaging the lexical network very strongly. On the other extreme, if a word is highly surprising, as represented at the top of Fig. 1, it takes a longer time to be processed. The upper corner of Fig. 1 seems to show some OSC modulation. Highly surprising words might require deep lexical processing, and a strong engagement of the lexical network. In this case, it is important whether a word’s orthography points to its meaning more or less strongly (i.e., has lower or higher OSC). In the case of intermediate Surprisal—arguably the most common situation in language comprehension (cf. Ferreira & Lowder, 2016)—there is room for readers to fully engage the ortho-lexical network, that is, to process the form–meaning connection. Low OSC implies a weaker cue toward a specific meaning and thus elicits longer processing times. High OSC, on the other hand, represents a strong cue for a very specific meaning; although this might determine a quicker identification of this specific word, there is also a greater potential for conflict with sentence-level expectations. This might happen, for example, when the word form points to a very specific meaning, which is, however, not the meaning that the sentence context points to. From this perspective, a word with intermediate OSC seems ideal—its form provides some indication for its likely meaning but is flexible enough to avoid conflicts with sentence-level constraints.

Of course, shorter gaze durations for intermediate levels of Surprisal may seem counterintuitive. However, this is only true if we assume that readers would ultimately *resolve* all ambiguity while fixating on the word, that is, they would always identify a word’s lexical-semantic content *precisely*. Readers do not necessarily need this level of precision, or at least not before they move on to the next word. They may well tolerate some preliminary uncertainty about the precise identity of the fixated word (cf. Levy et al., 2009). In fact, readers do seem to adopt a fuzzy processing strategy, balancing out processing time and precision. For example, for words with intermediate Surprisal, readers focus more on sentence-level meaning integration, rather than on precise lexical identification, at least until more evidence can be collected during fixations of subsequent words.

The pattern of results for right-bounded time and regression-path time is very similar to the one for gaze durations. The consistency of this interaction between different eye-movement measures speaks for a general trade-off between Surprisal and OSC that is robust over time and contributes to the integration of the fixated-word meaning into the sentence context. One deviation from the otherwise consistent pattern is first fixation duration, for which we only found a main effect of Surprisal, but no evidence for an interaction with OSC. Since first fixation reflects the very first encounter with a word, its duration is guided mostly by the preceding context (i.e., predictability) rather than the word itself (cf. Staub, 2011). The absence of an effect surely needs to be interpreted with caution but seems to suggest that the interplay of word- and sentence-level expectations emerges at somewhat later, more thorough stages of processing (as evidenced in gaze duration patterns).

As mentioned in the Introduction, the present study is exploratory in nature, and the observed variation in reading times is rather small. We also did not have strong predictions on the shape of the interaction; and, of course, its interpretation is quite tentative. Hence, the results beg for further evidence of the,  robustness of the observed phenomenon. To this end, in Experiment 2 we explore the effects of Surprisal and OSC in a different task and dependent measurenamely, self-paced reading data (Frank et al., 2013). This will put our observations to the test and check whether results generalize to other on-line metrics of sentence reading. More specifically, there are two fundamental aspects of the results of Experiment 1 that we are interested in Experiment 2: whether word-level and sentence-level prediction interact *in any way*, and whether they interact in such a way that intermediate levels of OSC and Surprisal determine quicker reading times.

## Experiment 2

### Methods

#### Data

For the self-paced reading, we again turn to data from Frank et al. (2013). In addition to the eye-tracking data analyzed above, these authors provide self-paced reading data from 117 university students (92 female, *M*_Age_ = 18.9 years) reading 361 English sentences, a superset of the sentences in the eye-tracking study. The sentences in their study were presented word by word and each word was replaced by the next one via key press of the participant (for details, see Frank et al., 2013). The time between word presentation onset and key press was measured as the reading time of that word. For our purposes, we only used reading times from the 205 sentences that were also used in the eye-tracking study because (1) this way the datasets are more comparable and (2) Surprisal values are only available for this subset in Frank et al. (2015). We used those Surprisal values and the OSC values (Marelli & Amenta, 2018) as in Experiment 1.

#### Analysis

Analysis of the self-paced reading data followed the same procedure as for the eye-tracking data in Experiment 1. First, again, we excluded data for which OSC values were not available from Marelli and Amenta (2018; 31.9%), and words for which OSC was equal to 1 (2.7%; following Marelli & Amenta, 2018). Next, data points with reading times shorter than 150 ms or longer than 1,500 ms were excluded (4.1%). The final dataset included 86,101 data points. We used GAMMs (Wood, 2006) to model log-transformed reading times. As in Experiment 1, the interaction between Surprisal and OSC of the fixated words was first modeled in nonlinear terms through tensor products. Word length, log-transformed frequency, and position in the sentence were included in the model as linear terms, and random effects for subjects and items were modeled through splines (Feldman et al., 2015). The model was then compared with the same model with a linear interaction to test whether nonlinearity is justified.

### Results

The analyses of self-paced reading times explained 40.6% of deviance and showed a significant nonlinear interaction between Surprisal and OSC (*p* < .0001). However, the inclusion of nonlinear terms was not supported in terms of a goodness-of-fit test: The amount of explained variance by a model including the nonlinear term compared with a model with a linear interaction was not significant. When modeled in linear terms, a significant interaction of Surprisal and OSC was still observed (*t* = 3.11, *p* = .0019): Goodness-of-fit comparison of a model with and without a linear interaction revealed that inclusion of the interaction was supported (*F* = 20.37, *p* < .001). The result held against model criticism (*t* = 2.98, *p* = .0029). Full details of this final model are reported in Table 5.

**Table 5:** Summary of the model fit to the log-transformed reading times

	Estimate	Std. Error	*t* value	Pr(>|t|)	*t* value after model criticism	Pr(>|t|) after model criticism
Intercept	5.63	0.027	212.42	<.001	212.06	<.001
Length	<−0.001	0.002	−0.27	.788	−0.17	.866
log-Frequency	<−0.001	<0.001	−0.08	.936	0.08	.936
Position in sentence	−0.001	<0.001	−3.38	<.001	−3.23	.001
OSC	−0.039	0.015	−2.50	.012	−2.45	.014
Surprisal	−0.003	0.002	−1.59	.112	−1.48	.138
OSC × Surprisal	0.006	0.002	3.11	.002	2.98	.003
	edf	Ref.df	*F*	*p* value	*F* after model criticism	*p* value after model criticism
s(Subject)	115.7	116	433.27	<.001	419.66	<.001
s(Word)	109.5	352	0.644	<.001	0.62	<.001

s() denotes a thin plate regression spline.

### Discussion

**Fig. 3:**
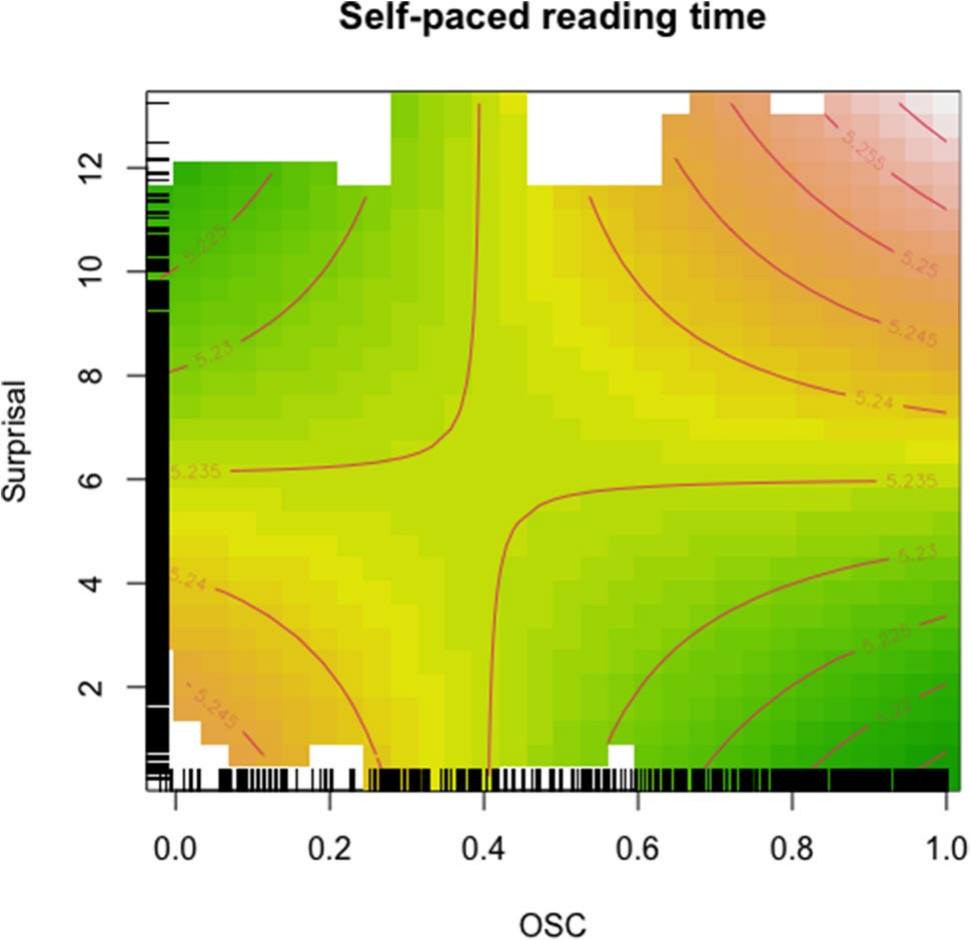
Interaction of OSC (*x*-axis) and Surprisal (*y*-axis). Color shades indicate log-transformed self-paced reading times (green are shorter times, red are longer times). The rugs indicate isochronous points. (Color figure online)

Results from Experiment 2 are based on the same material as Experiment 1, but with a different task and a different participant group. They confirm that sentence context and word orthography have a joint effect on comprehension in reading: we again found Surprisal and OSC to interact. In contrast to Experiment 1, however, we found no support for the nonlinearity of such an interaction. We found a linear pattern instead, which is characterized by longer reading times for words that are not expected given the context (high Surprisal), but whose word form is a strong cue for their meaning (high OSC; cf. upper right area in Fig. 3). Words with high Surprisal carry a lot of new information, which needs to be taken in. When the word also has high OSC, it provides a strong cue towards a specific word’s lexical-semantic content. In some cases, these strong expectations at the word level might conflict with the new and unexpected information at the sentence level, leading to longer self-paced reading times. This converges with Experiment 1: If a word is very unexpected from the sentence context (i.e., highly surprising), but that word strongly points to a specific meaning (high OSC), processing can be hampered. Shorter self-paced reading times are observed for words that are expected from sentence context (low Surprisal) and whose orthography consistently points to a certain meaning (high OSC): This combination results in easier processing, as represented by the green area in the bottom right of Fig. 3. Words with high Surprisal and low OSC also have shorter response times. While a highly surprising word might require deep lexical-semantic processing, low OSC means the word’s orthography does not point consistently toward any semantic content. If we assume, as in Experiment 1, that readers do not always immediately identify a word’s lexical-semantic content *precisely*, low OSC leaves them better opportunities than high OSC for such a fuzzy strategy. Hence, focusing on sentence-level meaning integration, rather than on precise lexical-semantic identification, allows for shorter response times, while more precise processing might still follow (even during the reading of subsequent words).^5^ This highlights again a general trade-off between sentence-level expectation and word-level orthographic cues.

## General discussion

In the present study, we sought to jointly examine two distinct but potentially interacting sources of information that readers can draw on when processing word meanings in sentences: sentence context and word orthography. We explored the effect of Surprisal and OSC on gaze durations, first fixation durations, right-bounded time, and regression-path time (Experiment 1), as well as self-paced reading times (Experiment 2). This brings research on word-level and sentence-level reading closer together. Our exploratory analyses provide first indications that Surprisal and OSC interact when reading words in sentences. Although the exact shape of this interaction remains a matter of future investigations, the observed pattern clearly indicates that how OSC “kicks in” depends on how surprising the word is given the sentence so far. In general, we clearly observe that reading times are longer when high Surprisal (i.e., word not expected from context) meets high OSC (i.e., meaning highly expected based on orthography). Reading times are shorter when they are both intermediate (Experiment 1) or one is high while the other is low (i.e., high Surprisal and low OSC or low Surprisal and high OSC; Experiment 2). In interpreting this, it is important to remember that the measures go in opposite directions, that is, *high Surprisal* means that the word was *lowly expected* given the context, whereas *high OSC* means that the meaning was *highly expected* based on the word form. There seems to be a general trade-off between sentence-level constraints and word-level cues in reading, in the sense that they can be best appreciated when their strength is balanced out, so that they are helpful, but not too restricting for getting to the meaning.

To illustrate the interaction between Surprisal and OSC, let us consider examples from the data. The word *has* features low values in both dimensions: It is not very surprising in the sentence *A horse has thrown a shoe*, and its orthography does not consistently point to one meaning because all its orthographic relatives (*hash, hashish, hassle, haste, hasty*) are semantically unrelated. The word *back* is comparably low in Surprisal in the sentence *He sighed and walked back to the wood shop*, but the OSC of *back* is rather high because its orthographic relatives are all semantically related (*backache, backbone, backdoor, backed, backer, backup,* etc.) and hence its orthography is very telling about its meaning. On the opposite side of the Surprisal spectrum are *sent* and *talk*. *Sent* is highly surprising in the sentence *Have a carriage sent for us immediately* and has low OSC with semantically unrelated orthographic relatives (*sentence, sentient, sentry*). *Talk* is similarly surprising in the sentence *I can't see any amount of talk getting you out of this mess*, but its orthography points very consistently to one meaning as all its orthographic relatives are also semantically related (*talker, talkie, talking*). An example for intermediate values of Surprisal and OSC (the middle ground in the gaze durations in Experiment 1) is *cup* in the sentence *He was already up and dressed and invited us in for a cup of tea* and with one semantically related and one unrelated orthographic neighbor (*cupboard, cupid*).

**Fig. 4:**
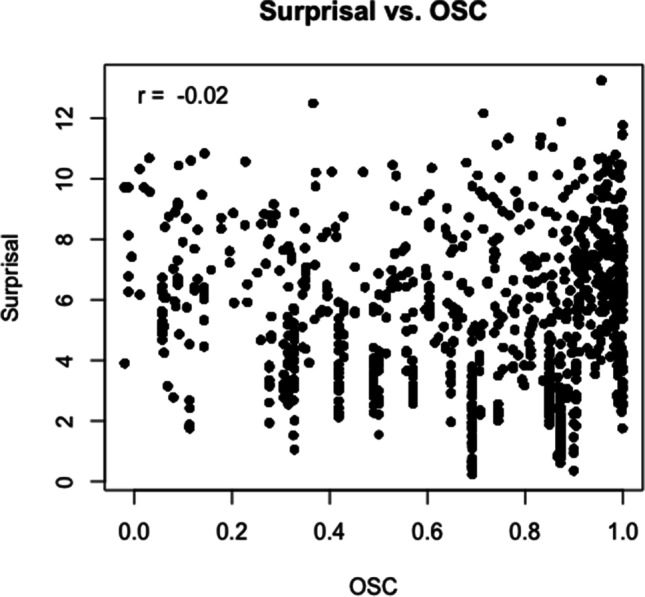
Scatterplot of Surprisal and OSC values in the dataset. The Pearson correlation coefficient is *r* = −.02

What becomes clear from these illustrative examples (see also Table 6) is that there is no obvious systematic confound with regard to the specific words at different levels of Surprisal and OSC that easily explains the observed pattern of reading times, i.e. there is no area of the distribution where words are particularly “weird” in any sense. Surprisal and OSC in our dataset also do not correlate (*r* = −.02, Fig. 4) and their distribution covers a great range of values (see also rugs in Figs. 1, 2 and 3). Moreover, the same words—hence having the same OSC values—can appear with different Surprisal values: *sent* also appears with intermediate surprisal in the sentence *It had been two weeks since Philip had been sent to prison* and so do *talk* (*You know better than to talk to your mother like that*) and *on* (*If I have time at the end I’ll fill you in on what happened*), indicating that specific words are not confounded with specific Surprisal values in our data.

**Table 6:** Examples for words at various levels of Surprisal and OSC

Surprisal	OSC		
	Low (<.2)	Intermediate (.3–.6)	High (>.8)
High (>9)	sent: sentence, sentient, sentry *Have a carriage sent for us immediately.*	mid: midday, midge, midget, midwife, midnight, midst^a^ *He rose from his seat and stopped mid way when Joe glared at him.*	talk: talker, talkie, talking *I can’t see any amount of talk getting you out of this mess.*
Intermediate (5–8)	tea: teabag, teatime, teacher, team, teaser, tear, teal^a^ *Finally Maria sat down with a cup of tea and a sandwich.*	cup: cupboard, cupid *He was already up and dressed and invited us in for a cup of tea.*	book: bookmark, bookshelf, bookworm, booklet, bookish^a^ *If this were a movie instead of a book this would be a good bit.*
Low (<3)	has: hash, hashish, hassle, haste, hasty *A horse has thrown a shoe.*	out: outage, outbreak, outcome, outsmart, ouzo^a^ *They’re riding out to meet them.*	back: backache, backbone, backdoor, backed, backer, backup^a^ *He sighed and walked back to the wood shop.*

The word of interest is first given in underlined font, followed by its orthographic relatives, and then the sentence in which it appears in italics.

^a^ Note that only a selection of orthographic relatives is given, as there were too many to list them all.

The slight differences between intermediate-intermediate and high-low combinations of surprisal and OSC in Experiment 1 and 2 might be due to differences in measurement granularity between the tasks. Eye tracking (even when considering later measures like regression-path time) measures more fine-grained and automatic mechanisms than self-paced reading, while the latter requires a more conscious decision to press a button and thus always involves additional cognitive mechanisms. A promising endeavor for the future would hence be to examine the exact time-course of the interaction.

An important implication concerns the relevance of the observed interaction for models of reading. Our study was motivated by a perceived gap between research on single word recognition and sentence processing. This gap is also reflected in models of reading that usually either explain eye movements in the reading of sentences (e.g., E-Z Reader; Reichle et al., 1998, 2003; SWIFT; Engbert et al., 2005) or the orthographic processing of single words (e.g., Coltheart et al., 2001; Norris, 2013; Rumelhart & McClelland, 1982, for an overview), but rarely consider both jointly (but see Snell et al., 2018). Hence, our study gives valuable indications for bridging models of sentence and word reading. If we had found an effect of Surprisal only, this would have suggested that sentence-level dynamics override word-level dynamics. This would have pressured models of orthographic processing to reconsider whether their assumptions hold for natural reading of sentences (vs. artificial, single-word laboratory settings). If we had found an effect only of OSC, this would have indicated that word-level dynamics dominate over sentence-level dynamics, suggesting that subtle word properties are much more important than previously thought. However, we found Surprisal and OSC to both impact word reading in sentences in an interactive way. This highlights that both variables have relevance for the reading process, and sentence-level and word-level processing needs to be better integrated in models of reading.

In conclusion, the present study represents the first attempt to investigate the interplay of two sources of information in reading: sentence-level context and word-level dynamics (form–meaning mapping). This investigation brings research on single-word and sentence reading closer together. We observed that both sources of information exert an influence on reading in an interactive way. There seems to be a general trade-off between the two such that high values in both (high Surprisal and high OSC) are detrimental, but when words provide intermediate amounts of evidence on both dimensions, initial processing is facilitated. While the shape of the interaction is not clear, we interpret the results as readers probably prioritizing coarse sentence-level meaning integration over precise lexical identification as a strategy for efficient language comprehension in reading. This highlights the need for models of sentence reading to consider factors of orthographic identification in more detail. Our explorative results open new opportunities for future research, especially confirmatory studies with more targeted hypotheses on the intersection of sentence context and word orthography as well as studies looking into the time-course of this interaction.

## Supplementary Information

ESM 1 (DOCX 98 kb)

## Data Availability

The datasets generated during and/or analyzed during the current study are available in the Open Science Framework (OSF) repository (https://osf.io/qaxje/).

## Footnotes

1 For a different characterization of the Orthography-Semantics Consistency, see Siegelman et al. (2022).

2 Experiment 1 results hold when considering the transformer-based measures by Merkx and Frank (2021).

3 Depending on the adopted semantic model, negative values of OSC are theoretically possible. However, practically speaking these cases are rare, and typically close to 0. Further considerations in this respect are provided in Marelli and Amenta (2018).

4 Additionally including previous word’s frequency as a fixed effect in order to control for spillover effects does not change the overall pattern of results.

5 Indeed, additional analysis of RT on subsequent words, presented in the Supplementary Material, did yield some indication of spillover effects.
