# Supplementary material for: Prediction at the intersection of sentence context and word form: Evidence from eye-movements and self-paced reading
Source: Psychon Bull Rev. 2022 Dec 12;30(3):1081–92. doi: 10.3758/s13423-022-02223-9 (PMC10264485; doi:10.3758/s13423-022-02223-9)
Supplement: Supplementary file 1 — (DOCX 98 kb) [file 13423_2022_2223_MOESM1_ESM.docx]

**Supplementary Material**

In the following, we report additional analyses for Experiment 2 focused on potential spillover effects. Self-paced reading times have been shown to be sensitive to spillover effects, and much more so than eye tracking measures. It is hence possible, as mentioned in the Discussion section of Experiment 2, that some aspects of processing are delayed. For example, readers might not always identify a word’s lexical–semantic content *precisely* while reading that word; more precise processing might become evident only during the reading of subsequent words. To explore this possibility, we ran additional analyses on the self-paced reading times of the next word (*t+1*) and the word thereafter (*t+2*).

For the additional analyses, we followed the same modeling procedure as in the other analyses, using Generalized Additive Mixed Models (GAMMs; Wood, 2006) including the interaction between surprisal and OSC of word *t* as non-linear terms, and length, log-transformed frequency, and position of word *t* in the sentence as linear terms, as well as random effects for subjects and items. Importantly, for these analyses, the dependent variables were log-transformed reading times on word *t+1* and *t+2* , instead of *t*.

Results of the analysis on RT at *t+1* are reported in Table S1. We observed a significant non-linear interaction between Surprisal and OSC (*p*<.0001). The inclusion of non-linear terms was justified by a goodness-of-fit test: the fit of the model with the tensor product was significantly higher than the one obtained when the interaction was modeled in linear terms (*F*=3.17, *p*=.043). The non-linear interaction held against model criticism.

Table S1. Summary of the model fit to the log-transformed RT of *t+1*. te() denotes a tensor smooth, s() denotes a thin plate regression spline.

|  | Estimate | Std. Error | t-value | Pr(>\|t\|) |
| --- | --- | --- | --- | --- |
| Intercept | 5.59 | 0.026 | 214.52 | <0.001 |
| Length | <-0.001 | 0.002 | -0.11 | 0.916 |
| log-Frequency | <0.001 | <0.001 | 0.29 | 0.773 |
| Position in sentence | 0.002 | <0.001 | 4.84 | <0.001 |
|  | edf | Ref.df | F | p-value |
| te(OSC, Surprisal) | 5.49 | 6.74 | 9.72 | <0.001 |
| s(Subject) | 115.73 | 116.00 | 433.53 | <0.001 |
| s(Word) | 117.57 | 352 | 0.653 | <0.001 |


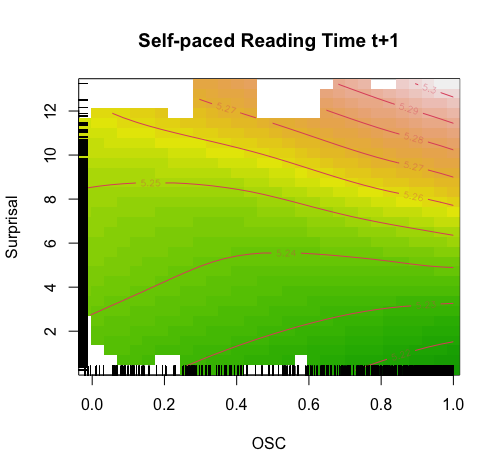


Figure S1. Tensor product smooth for the interaction of OSC (x-axis) and Surprisal (y-axis), color shades indicate different log-transformed self-paced reading times at t+1 with green shades indicating shorter times and red shades indicating longer times, rugs indicate distribution of data points.

In a second step, we also included the predictors of *t+1*, that is, Surprisal of *t+1*, OSC of *t+1*, length of *t+1*, frequency of *t+1*. We did so because, obviously, RTs to words *t+1* should also be influenced by their own OSC and surprisal values. Even with this extended control analysis, the interaction still survives (*F*=7.08, *p*>0.001). However, in this analysis, we lose a drastic amount of data points: not only all the data points with missing OSC values of word *t*, but also those with missing OSC values of word *t+1* (44,156 data points instead of 86,101 in the original analyses as reported in the main manuscript).

We ran the same set of analyses to model RT at *t+2*. For this, the evidence for a spillover interaction turned out weaker: non-linearity was not supported by the model comparison (*F*=1.90, *p*=0.09). If included as a linear interaction, it was significant (*F*=2.02, *p*=0.04). These results are reported in Table S2 and depicted in Figure S2.

Table S2. Summary of the model fit to the log-transformed RT of *t+2*. s() denotes a thin plate regression spline.

|  | Estimate | Std. Error | t-value | Pr(>\|t\|) |
| --- | --- | --- | --- | --- |
| Intercept | 5.59 | 0.09 | 193.67 | <0.001 |
| Length | -0.001 | 0.002 | -0.62 | 0.533 |
| log-Frequency | <0.001 | <0.001 | 0.749 | 0.454 |
| Position in sentence | 0.002 | <0.001 | 3.70 | <0.001 |
| OSC | -0.029 | 0.019 | -1.50 | 0.134 |
| Surprisal | <0.001 | 0.002 | 0.413 | 0.680 |
| OSC x Surprisal | 0.005 | 0.002 | 2.021 | 0.043 |
|  | edf | Ref.df | F | p-value |
| s(Subject) | 115.7 | 116 | 388.34 | <0.001 |
| s(Word) | 157.4 | 329 | 1.196 | <0.001 |


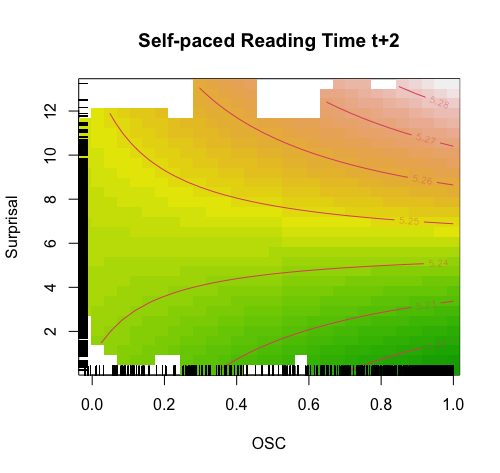


Figure S2. Tensor product smooth for the interaction of OSC (x-axis) and Surprisal (y-axis), color shades indicate different log-transformed self-paced reading times at *t+2* with green shades indicating shorter times and red shades indicating longer times, rugs indicate distribution of data points.

When we again used the fuller model for RT at *t+2*, including predictors for word *t+1* and *t+2* as well, there was no reliable evidence for an interaction effect of surprisal and OSC: while the non-linear interaction emerged significant (F=2.79, p=0.013), model comparison did not support inclusion of the non-linear term. A linear interaction was not significant (F=1.74, p=0.082) and its inclusion was also not supported by model comparison (F=0.59, p=0.697). Very notably, the dataset was drastically reduced here with 26,288 data points (vs. 86,101 in the original analysis).

While these additional analyses do show some indication for spillover / delayed effects of Surprisal and OSC, the results should be taken with a grain of salt, since the dataset they are based on is very reduced in size because not only data points with missing values at word *t* but also at *t+1* (and *t+2*) had to be excluded, obviously. This makes the additional analyses less reliable and less comparable to the original analysis of word *t*.
